# The positive education of challenge: innovative integration of challenge based learning and positive education

**DOI:** 10.3389/fpsyg.2023.1225122

**Published:** 2023-12-04

**Authors:** Keisuke Horikoshi

**Affiliations:** Institute of Interdisciplinary Research, Kyoto University of Advanced Science, Kyoto, Japan

**Keywords:** positive education, challenge based learning, challenge, well-being, positive psychology, challengership education, integration

## Abstract

Challenge based learning is not generally considered part of positive education. This study argues that challenge based learning should be considered and integrated with positive education to advance it from three perspectives. First, the aims of both educational approaches emphasize the promotion of engagement, meaning, achievement, and learning. Second, empirical studies suggest that challenge based learning is likely to enhance well-being and learning outcomes, aligning with positive education’s impact. Third, incorporating challenge based learning in positive education will likely address multiple criticisms of positive education and help advance it by incorporating real-life challenges, meaningful contexts, experiential learning, collective perspectives, and educational studies. To advance positive education, more empirical studies should be conducted on the impact of challenge based learning on well-being to fill the research gap in quantity, scope of variables, and longitudinal studies, with due consideration of its variety in implementation. Furthermore, innovative integration of challenge based learning and existing positive education interventions should be explored based on comparative analyses of both approaches to develop positive education that enables learners to flourish with challenges at the individual and collective levels. Positive education that explicitly incorporates challenges is called the positive education of challenge. To advance the positive education of challenge, more educational approaches (other than challenge based learning) that focus on challenges (e.g., challenge courses and pedagogy of challenge) should also be explored for integration with positive education.

## 1 Introduction

Since Seligman et al. (2009) conceptualized positive education (PE) in 2009, there have been many studies and practices of PE with the advancement of positive psychology (PP), in which the effectiveness of PP/PE interventions in school settings have been examined (e.g., Shankland and Rosset, 2017; Waters and Loton, 2019). At about the same time, since an educational method called challenge based learning (CBL) was developed through the initiative of Apple Inc., studies and practices of CBL have been growing (Leijon et al., 2022; Gallagher and Savage, 2023), while it has not been generally considered as a part of PE.

This study proposes the exploration of an innovative integration of CBL and PE. This study is intended for those who are interested in developing a better educational approach that enables learners to thrive with challenges; an approach needed in a rapidly changing world (e.g., OECD, 2019; Horikoshi, 2023). Because both CBL and PE have been considered relevant for a wide range of school settings including primary, secondary, and higher education (e.g., Seligman et al., 2009; Johnson and Adams, 2011; Nichols et al., 2016; Waters and Loton, 2021), the arguments on the integration of both approaches presented herein should also be relevant for those concerned with improving the aforementioned forms of education.

## 2 CBL

Apple Inc. developed CBL with a concern that traditional pedagogy was becoming ineffective in engaging students without incorporating sufficient real-world contexts (Nichols and Cator, 2008). CBL (Nichols and Cator, 2008, p. 1) is “collaborative and hands-on, asking students to work with peers, teachers, and experts in their communities and around the world to ask good questions, develop deeper subject area knowledge, accept and solve challenges, take action, and share their experience.” It incorporates elements of experiential learning (The Challenge Institute, n.d.a), where meaningful real-life challenges are identified based on big ideas or themes (e.g., sustainability, identity, and creativity) for learners to answer or solve in teams (Nichols and Cator, 2008). Studies and practices of CBL have spread around a wide range of countries, disciplines, and ages (e.g., Johnson and Adams, 2011; Leijon et al., 2022).

## 3 CBL should be considered as a part of PE and be integrated with PE to advance PE

This section examines the aim and impact of CBL in the PE context and outlines the advantages of integrating both approaches.

### 3.1 The aim of CBL is in line with that of PE

Seligman et al. (2009) describe PE as teaching “both the skills of well-being and the skills of achievement” (p. 294) and emphasize that it promotes better learning, where the elements of well-being include positive emotion, engagement, and meaning.

Challenge Based Learning White Paper (Nichols and Cator, 2008) describes the original aim of developing CBL as “engaging more students to achieve” (p. 1) and emphasizes the promotion of engagement, meaning, and importance for students and society. Teachers and students are advised to work on themes and challenges (Nichols and Cator, 2008, p. 5) that are “important and meaningful to their specific context.” The Challenge Institute (n.d.b) summarizes that CBL helps acquire knowledge and the skills “necessary to thrive in an ever-changing world.”

The comparison of the aims of PE and CBL makes it clear that the two approaches share an emphasis on promoting engagement, meaning, achievement, and learning. Therefore, the aim of CBL should align with that of PE.

### 3.2 Empirical evidence suggests that the positive impact of CBL is in line with that of PE

Although limited, there have been some empirical studies on the impact of CBL on wellbeing-related variables: (1) Regarding engagement, Johnson and Adams (2011) suggest that CBL effectively promotes students’ engagement in learning. Similarly, Simón-Chico et al. (2023) found that behavioral engagement (Fredricks et al., 2004; Skinner et al., 2008) in the CBL condition in physical education was higher after the intervention than before. (2) A study by Johnson and Adams (2011) suggests that CBL programs effectively enhance students’ social skills. López-Fernández et al. (2020) reported improvements in the relationship between teachers and students after they worked collaboratively to solve the challenges in their CBL program. (3) Regarding basic psychological needs (Chen et al., 2015), Simón-Chico et al. (2023) reported improvements in autonomy, competence, and relatedness satisfaction after a CBL program.

From the experiential learning perspective (e.g., Kolb, 1984), a meta-analysis suggests that experiential learning effectively enhances well-being (Chan et al., 2021). Since CBL incorporates elements of experiential learning (The Challenge Institute, n.d.a), it is likely to be effective in enhancing well-being, aligning with the aforementioned studies’ results.

Studies have also suggested that CBL effectively enhances achievements and learning skills. For example, studies have suggested that CBL effectively promotes learning (e.g., Johnson et al., 2009; Johnson and Adams, 2011; Simón-Chico et al., 2023). Improvements in leadership, critical thinking, creativity, problem-solving, and entrepreneurship have also been suggested (Johnson and Adams, 2011; Colombelli et al., 2022).

Although these are limited and incomprehensive studies of the impact of CBL, they suggest that CBL is likely to be effective in improving well-being and learning outcomes and, therefore, aligns with PE regarding its positive impact.

### 3.3 CBL is important to advance PE by incorporating real challenges in PE

Scholars have suggested that real challenges are not sufficiently incorporated in PE and should be incorporated to enable students to embrace and flourish with them. For example, White and Buchanan (2017) argued that PE needs to incorporate real challenge solving to better prepare students to deal with real-life challenges and thrive. Similarly, Ledertoug and Paarup (2021) emphasized the necessity for education to prepare students to embrace future challenges. In this context, the CBL’s focus on providing meaningful challenges matches these needs.

From a broader perspective, scholars have suggested integrating challenges in PP studies (e.g., Boniwell, 2012; Wong, 2016; Horikoshi, 2023). From the viewpoint of second wave PP, which emphasizes the dialectical integration of positive and negative elements (Wong, 2011; Lomas and Ivtzan, 2016), Horikoshi (2023) highlights the importance of challenges that often encompass the positive and negative elements of life.

In the context of the well-being and learning that PE aims to promote, studies on intrinsic motivation, curiosity, mindset, desirable difficulties, and goal setting emphasize the importance of challenges (e.g., Latham and Locke, 1991; Ryan and Deci, 2000; Kashdan and Silvia, 2009; Dweck and Yeager, 2019; Bjork and Bjork, 2020; Horikoshi, 2023). In similar contexts, education scholars argue for the significance of incorporating challenges in pedagogy (e.g., Higham et al., 2010; Winstanley, 2010).

The importance of incorporating real challenges into PE can be argued from the multiple theoretical frameworks of PP that focus on the relationship between well-being, challenges, and resources/skills. For instance, Csikszentmihalyi’s (2003) model of flow highlights the crucial role of a balance between challenges and skills as one of the conditions of flow, where flow is an extremely high level of engagement and an important element of well-being (Seligman, 2011). Hendry and Kloep (2002) theorized about human development from the perspective of the interplay between challenges and resources. Partially built on the essence of these works, Dodge et al. (2012, p. 230) defined well-being as “the balance point between an individual’s resource pool and the challenges faced.” In this framework of well-being as a challenge-resource balance (CRB), the processes of dialectical interactions between challenges and resources/skills are central to well-being, development, and learning, in which both challenges and resources/skills play unique and indispensable roles (Hendry and Kloep, 2002; Csikszentmihalyi, 2003; Dodge et al., 2012).

The CRB framework can be used to position the relationship between PE and CBL within a single framework. First, many existing PE programs focus on enhancing resources/skills related to well-being. For example, the original conceptualization of PE by Seligman et al. (2009, p. 294) states that PE aims to “teach both the skills of well-being and the skills of achievement,” and many interventions used in PE directly focus on building relevant skills (e.g., those for emotion, stress coping, and resilience: Waters and Loton, 2019) which can be transformed into resources to thrive with challenges. Second, CBL focuses on providing meaningful experiences of challenges conducive to well-being and learning, through which the development of relevant resources and skills is expected to be stimulated. Therefore, many existing PE programs can be interpreted as an approach that focuses on the resource/skill side, whereas CBL can be interpreted as an approach that focuses on the challenge side within the CRB framework of well-being. Because dialectical interactions between challenges and resources/skills are essential for well-being and development in the CRB framework, both educational approaches are indispensable where the elements of both approaches interact dialectically and function integrally.

From this perspective, the relationship between the two educational approaches should theoretically be considered complementary to mutual improvement. However, PE and CBL are generally practiced and studied separately. Therefore, their integration should be explored for mutual development, and the CRB framework can be a basis for the conceptual integration. Specifically, CBL is likely to complement PE by providing real challenges that are absent in many existing PE programs. On the other hand, the existing elements of PE are likely to complement CBL by directly and systematically teaching evidence-based knowledge and skills associated with dealing with challenges (e.g., stress coping, resilience, self-regulated learning, and strengths: Waters and Loton, 2019) that are not systematically taught in CBL to support students’ learning in challenging experiences.

### 3.4 CBL is important to advance PE by incorporating contexts, experiential learning, collective perspectives, and educational studies in PE

Incorporating CBL into PE is likely to advance PE by providing relevant contexts, collective/systems perspectives, and educational studies. First, scholars criticize PE for underemphasizing the importance of context in well-being and argue that it should incorporate appropriate context (Ciarrochi et al., 2016; Waters, 2021). Since CBL is designed to present students with engaging and meaningful contexts through real challenges (Nichols and Cator, 2008), it is well-positioned to address this important criticism.

Second, scholars have suggested that incorporating experiential learning into PE will likely improve its impact (Biswas-Diener and Patterson, 2011; Payne et al., 2020). Combining CBL with PE brings about challenges in PE, including elements of experiential learning (The Challenge Institute, n.d.a), and is likely to improve PE.

Third, scholars criticize PE for overemphasizing individualistic and underemphasizing collective (Trask-Kerr et al., 2019) and systems perspectives (Kern and Taylor, 2021) regarding well-being. In this context, CBL is structured such that students collaborate in teams, schools, and communities to solve important problems for students and society and take real action toward a better community (Nichols and Cator, 2008). Therefore, CBL is well suited to address this issue, emphasizing well-being not only at the individual level but also at the collective and system levels.

Fourth, scholars criticize PE as relying solely on psychology and not fully integrating studies and practices of other fields, including education (Trask-Kerr et al., 2019; White, 2021). Because CBL is a multidisciplinary approach (Nichols and Cator, 2008) that originated from educational practices and studies, it can contribute to PE by incorporating valuable studies and effective educational practices. Incorporating CBL into PE is also important from the perspective of third wave PP, which emphasizes an interdisciplinary approach (Lomas et al., 2021).

## 4 Research gap and future directions

### 4.1 More empirical research should be conducted on the impact of CBL from the PE perspective

Because CBL is not within the scope of PE, there are only a few studies on its impact on well-being-related variables (e.g., Johnson et al., 2009; Johnson and Adams, 2011; Bisanz et al., 2019; Pinho et al., 2019; López-Fernández et al., 2020; Franco et al., 2023; Simón-Chico et al., 2023). Therefore, more empirical research should be conducted on the impact of CBL on well-being to fill the research gap in terms of quantity, the scope of variables, and longitudinal studies with due consideration of the variety and modification of CBL.

Regarding the scope of impact, the scope of well-being-related variables measured in CBL research is limited compared with those in PE studies. For example, at the individual level, engaging in solving meaningful challenges through CBL in a team with a teacher’s support may be beneficial in promoting a variety of well-being-related variables, including a sense of meaning and life satisfaction, as well as in reducing negative symptoms (e.g., depression and anxiety) that are often measured in empirical studies of PE (e.g., Seligman et al., 2009; Waters and Loton, 2019). However, the impact of the CBL on these variables has not yet been examined empirically. Furthermore, the impact of CBL on well-being at the collective level has not yet been systematically examined from the perspective of PE.

In terms of longitudinal studies, existing ones on the impact of CBL on well-being tend to be measured at two time points (pre- and post-intervention) and lack data on how the impact changes or lasts over time. Therefore, longitudinal studies on this impact should be conducted.

Furthermore, because the implementations of CBL include a variety of applications and modifications without sufficient standardization (Gallagher and Savage, 2023; Van den Beemt et al., 2023), it is important to measure the impact of CBL that is more generalizable considering these different applications and modifications.

### 4.2 Innovative integration of CBL and existing PE interventions should be explored based on comparative analyses

As the characteristics of CBL (including its relative strengths and weaknesses) as a PE intervention have been left unexamined, its effectiveness should be systematically analyzed and compared with those of existing PE interventions based on empirical data. This comparison may include the scope of variables enhanced by the interventions (e.g., individual/collective well-being, skills for well-being, academic performance, other skills, and social impact), effect sizes, and lengths of effects.

One of the hypothetical characteristics of CBL as a PE intervention may be its potential benefits for collective well-being and social impact, considering its clear social orientation. Another possible characteristic is that it may effectively enhance a set of important skills to thrive under challenges (e.g., social skills, leadership, creativity, problem-solving skills, and entrepreneurship: Johnson and Adams, 2011; Colombelli et al., 2022).

Based on these comparative analyses, the selection and combination/integration of CBL and existing PE interventions should be explored to serve stakeholders’ specific needs better. For example, Waters and Loton (2019) identified 12 categories of representative PE interventions based on a literature review (e.g., strength-related, emotional intelligence, gratitude, meditation, mindfulness, mentoring, coping, resilience, self-regulated learning, and goal interventions). In line with the essence of the CRB framework discussed earlier, the synergistic combination/integration of CBL and one or more of these representative PE interventions should be explored to create better PE interventions.

In this context, there are limited cases that explicitly include elements of both CBL and some PE elements in the 12 categories identified by Waters and Loton (2019), which can be found in scientific journals published in English. Specifically, the Youth Start Entrepreneurial Challenges (YSEC: Lindner, 2018; Bisanz et al., 2019; Youth Start Entrepreneurial Challenges, n.d.) is a comprehensive educational program composed of a package of multiple approaches, including elements of CBL, social entrepreneurship education, education for sustainable development, holistic learning, and PE (including mindfulness, strengths, and empathy). The Youth Start Social Entrepreneurship Program for Kids (UKids) is a similar program built on parts of the YSEC (Pinho et al., 2019; UKids, n.d.). Bisanz et al. (2019) suggested that the YSEC effectively enhances social skills, self-efficacy, self-esteem, learning, and entrepreneurship. Pinho et al. (2019) suggested the benefits of UKids in supporting students’ social skills, self-confidence, and creativity. Some CBL programs also incorporate mentoring (e.g., Castro and Zermeño, 2020; Colombelli et al., 2022) to support student learning. However, these studies did not test and compare their programs with programs without PE elements to evaluate how adding certain PE elements changes their outcomes or what synergies emerge comprehensively from the perspective of PE. Therefore, further empirical studies of CBL that are combined with the PE interventions of mindfulness, strengths, emotional intelligence, or/and mentoring are needed.

In addition to these existing programs, there are many more possibilities for exploring the innovative integration of CBL and PE interventions, including combining CBL with one or more of the 12 categories identified by Waters and Loton (2019). For example, a combination of CBL and the PE of stress coping, resilience, or/and goal interventions should be explored. The studies on stress coping, resilience, and goal setting indicate that skills associated with these themes are helpful to thrive with challenges (Lazarus and Folkman, 1984; Latham and Locke, 1991; Reivich and Shatté, 2002; Horikoshi, 2023). In the combined programs, students can learn skills associated with stress coping, resilience, or/and goals through the elements of PE interventions and test/develop them through the real challenges provided by the CBL elements. Further studies of such programs that combine the elements of CBL and PE, functioning in a complementary and synergistic manner, can create a basis for the conceptual and empirical integration of both approaches in line with the CRB framework.

## 5 Discussion

The integration of CBL and PE aims to create better PE interventions that effectively equip learners to flourish with challenges by incorporating the experiences of real challenges in meaningful contexts at both the individual and collective levels. PE that explicitly incorporates challenges can be called the PE of challenge. The PE of challenge is also an education for challengership (“activities and processes involving challenges”: Horikoshi, 2023).

### 5.1 More educational approaches that focus on challenges should be explored for integration with PE to advance the PE of challenge

In addition to CBL, additional educational approaches that explicitly focus on challenges should be explored and examined from the perspective of PE to advance the PE of challenge and challengership education. One possibility is a challenge course (CC) program, which usually includes outdoor group activities with physical and mental challenges and can be adapted to indoor settings (DuFrene et al., 1999; Gillis and Speelman, 2008). A meta-analysis on the impact of CC suggested its benefits in enhancing self-efficacy and group dynamics (Gillis and Speelman, 2008). Snyder (2015) argued that CC and positive youth development (Romer and Hansen, 2021) share significant similarities and that their integration should be explored. In another instance, Higham et al. (2010) and Higham (2016) argued that deliberately incorporating real contextualized challenges in pedagogy is conducive to the education of responsible leadership (a concept partially based on authentic leadership: Avolio and Gardner, 2005) and conceptualized this approach as a pedagogy of challenge (POC). The results of a POC program suggest its benefits in enhancing confidence, resilience, teamwork-related skills, and academic performance (Higham et al., 2010) and can be considered in line with those of PE. Further studies are needed to evaluate their impact in the context of PE and to explore their integration with PE to advance the PE of challenge.

In summary, this article argues that CBL should be integrated with PE and that more educational approaches that explicitly focus on challenges should be explored for integration with PE to advance the PE of challenge.

## Data availability statement

The original contributions presented in the study are included in the article/supplementary material, further inquiries can be directed to the corresponding author.

## Author contributions

The author confirms being the sole contributor of this work and has approved it for publication.
